# Supplementary material for: Anisotropy in mechanical unfolding of protein upon partner-assisted pulling and handle-assisted pulling
Source: Commun Biol. 2021 Jul 29;4:925. doi: 10.1038/s42003-021-02445-y (PMC8322310; doi:10.1038/s42003-021-02445-y)
Supplement: Supplementary file 2 — Supplementary Information [file 42003_2021_2445_MOESM2_ESM.pdf]

Anisotropy in mechanical unfolding of protein upon partner-assisted pulling and handle-assisted pulling

Nisha Arora[a], Jagadish Prasad Hazra[a,✉], Sabyasachi Rakshit[a,b,✉]

[a]Department of Chemical Sciences, Indian Institute of Science Education and Research Mohali, Punjab, India

[b]Centre for Protein Science Design and Engineering, Indian Institute of Science Education and Research Mohali, Punjab, India

Figure 1:	SDS-PAGE gel and western blot pictures	3
Figure 2:	Mean number of unfoldings in each force curve	4
Figure 3:	Unfolding force distributions of Cdh23 in both HAP and PAP mode of force application	5
Figure 4:	Force distributions for initial stretches in both HAP and PAP mode	6
Figure 5:	Estimating kinetic parameters of unfoldings considering apparent loading rate measured from linear slopes of force-extension curves immediate prior to unfolding or unbinding	7
Figure 6:	Free energy changes for different types of unfolding	8
Figure 7:	Spatial positions of the three major interfacial nodes	9
Supplementary Tables 1 and 2:	Kinetic parameters for unfolding of Cdh23 EC1-27 in PAP and HAP mode	10

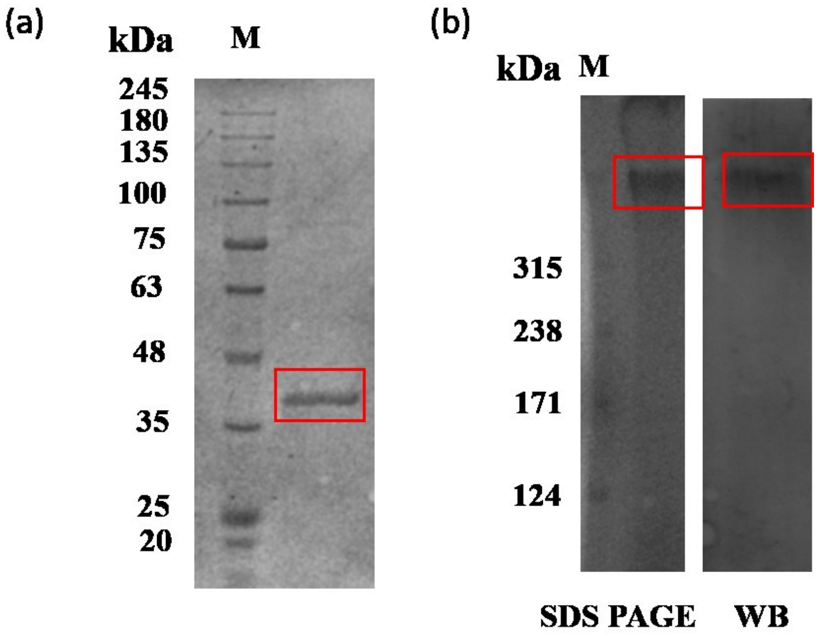

Supplementary Figure 1. SDS-PAGE and western blot of purified proteins. We have expressed Cdh23 EC1-27 and Pcdh15 EC1-2 using mammalian Expi-CHO cell lines and protein expression has been confirmed using SDS-PAGE gel as well as western blot. (a) 12% SDS-PAGE gel picture for Pcdh15 EC1-2. (b) 6% SDS-PAGE gel (left) and western blot (right) pictures of Cdh23 EC1-27. Lane 'M' represents marker and red box indicates the desired protein bands. Western blot was performed using antibody against the ectodomains of Cdh23 (PA5-43398, Thermo Fischer Scientific).

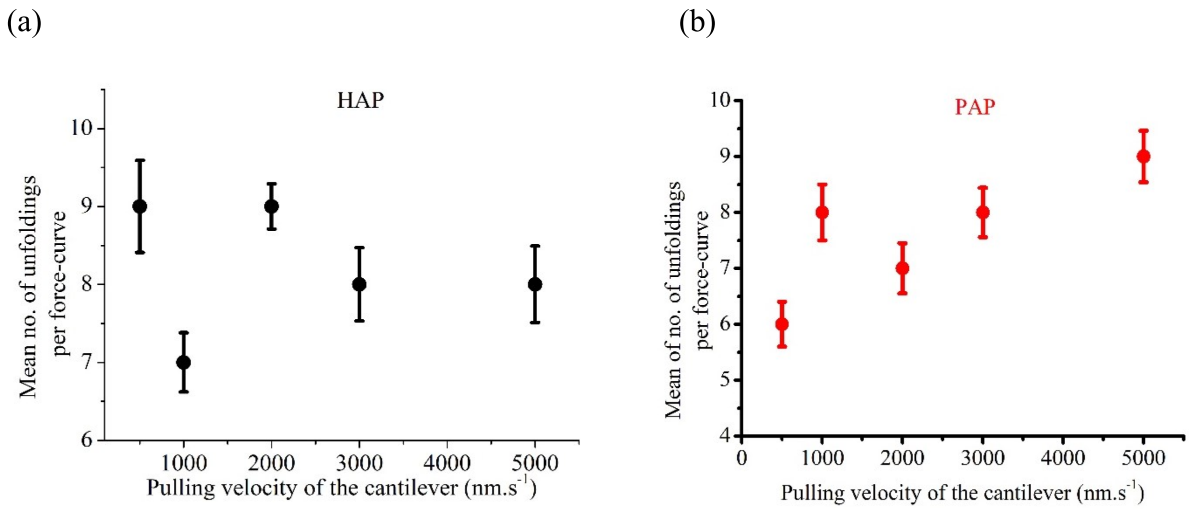

Supplementary Figure 2: Unfolding numbers per force curves. (a) The average number of unfoldings of Cdh23 EC1-27 per force curve is shown for HAP in a scattered plot. Error bars denote the standard error of the mean (SEM), where n ranges between 40-70**.** (b) An average number of unfoldings of Cdh23 EC1-27 per force curve is shown for PAP in a scattered plot. Error bars denote the standard error of the mean (SEM), where n ranges between 40-70.

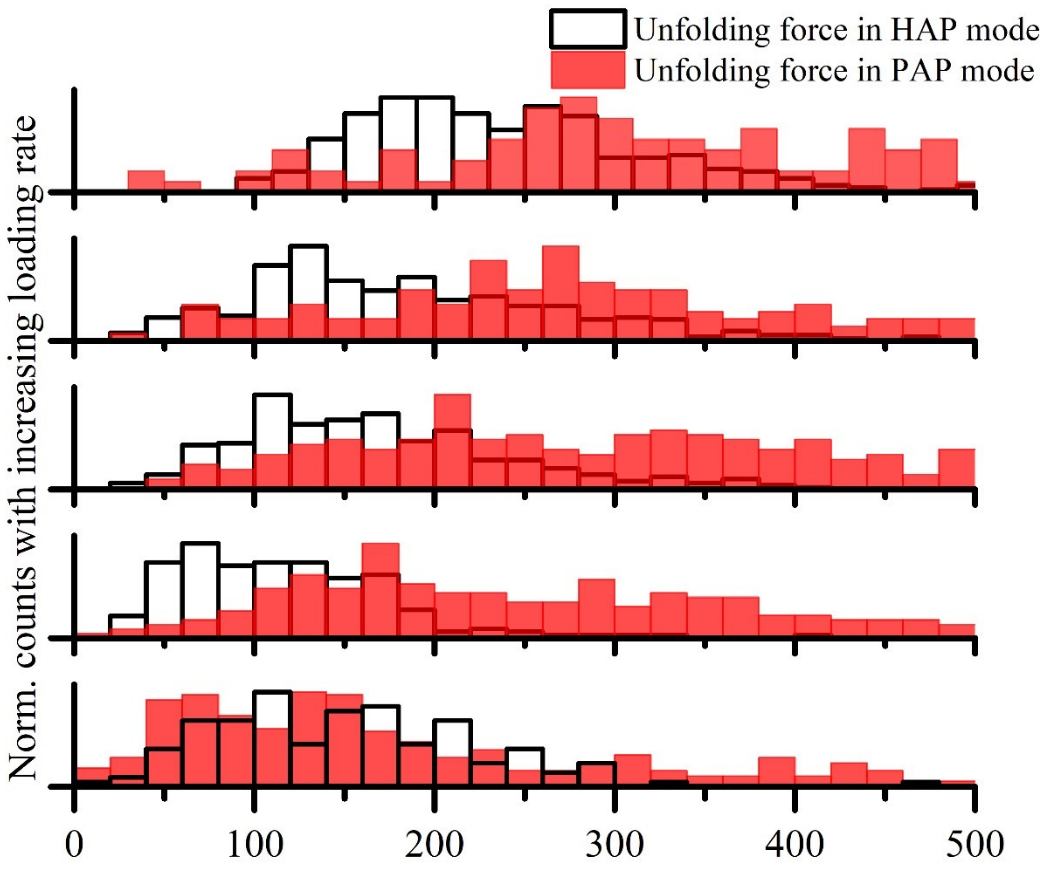

Supplementary Figure 3. Distributions of unfolding forces of Cdh23 in HAP and PAP (In support of figure 1). Unfolding force distributions of Cdh23 in HAP (open black box, n=838) and PAP (red filled box, n = 946) modes at various pulling velocities of 500, 1000, 2000, 3000, 5000 nm.s^{-1}. PAP mode of pulling registers higher forces of unfolding compared to HAP.

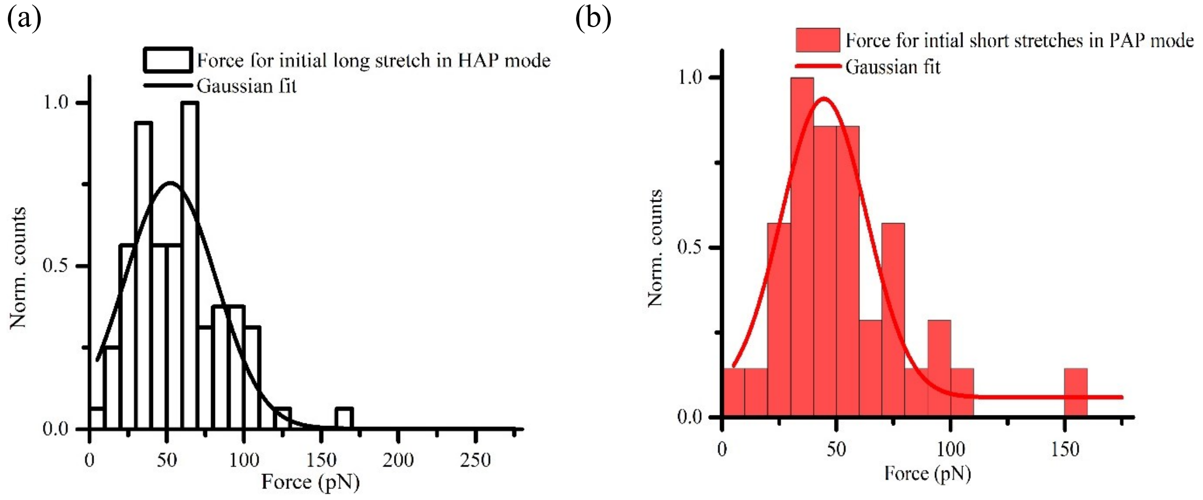

Supplementary Figure 4. Forces for initial length gain in HAP and PAP mode. (In support of figure 1) (a) In HAP, we noticed an initial extension of 75.0±2.2 nm in force curves. The corresponding force distribution obtained for this long extension at 2000 nm s^{-1} pulling velocity is shown here. The peak force is 52.4±3.2 pN. (b) Contrary to HAP, a small initial stretch of mean length of 6.5±0.3 nm is observed in PAP. The corresponding force distribution obtained for this short extension at 2000 nm s^{-1} pulling velocity is shown here. The peak force is 44.6±2.4 pN.

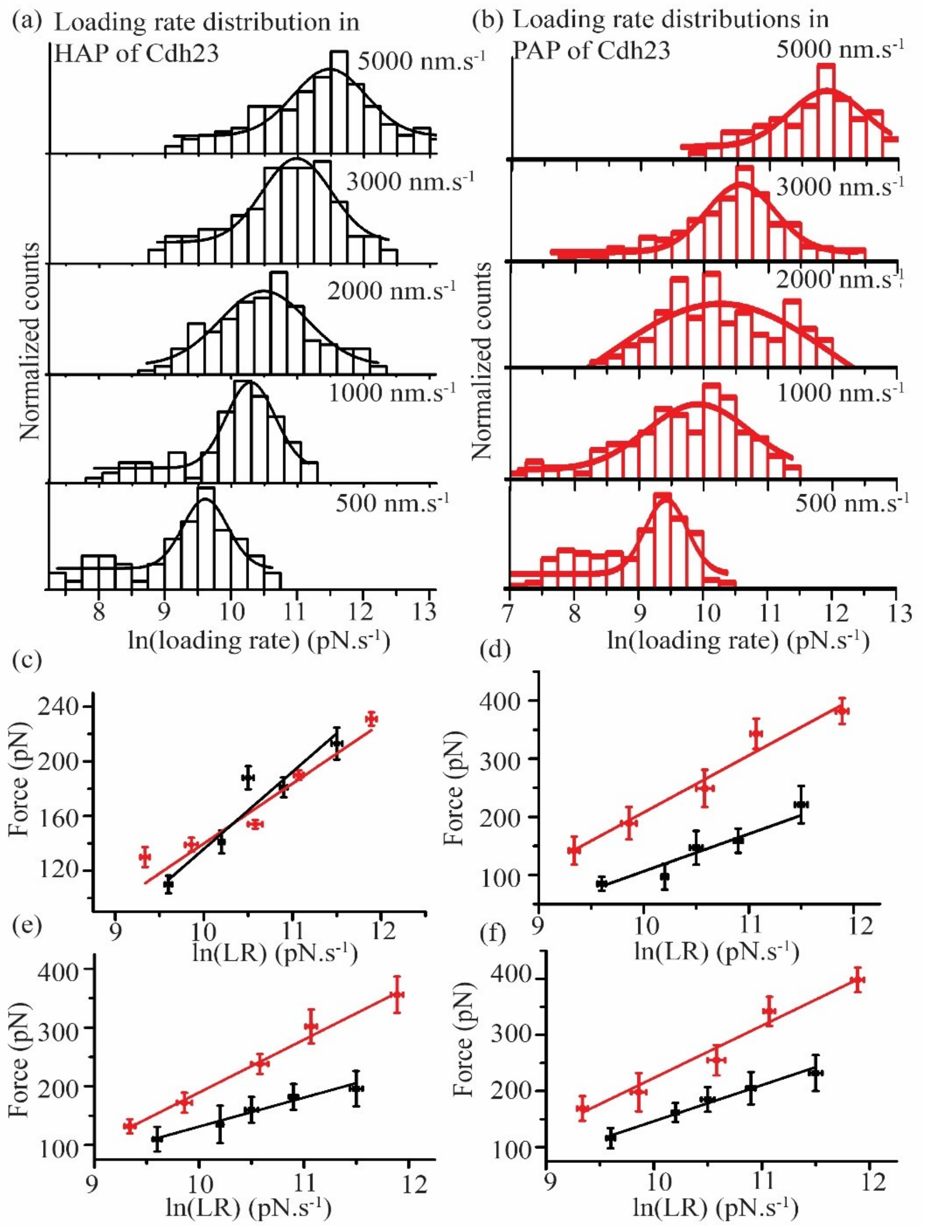

Supplementary Figure 5: Estimation of kinetic parameters of unfoldings considering apparent loading rate measured from linear slopes of force-extension curves immediate prior to unfolding or unbinding: (a) Histograms of loading rates determined from the slopes of the linear portion of force-extension curves obtained in HAP of Cdh23 EC1-27. (b) Histograms of loading rates calculated from the slopes of the linear part of force-extension curves obtained in PAP of Cdh23 EC1-27. Loading rates were estimated by multiplying the slope of the curves with the corresponding pulling velocities. Histograms are fit to the Gaussian peak function. (c-f) Most-probable unfolding forces are plotted with the experimentally determined apparent loading rates for HAP and PAP of Cdh23 EC1-27 for different extension types of (c) ~6.5 nm, (d) ~16 nm, (e) ~24 nm, and (f) ~33 nm, respectively.

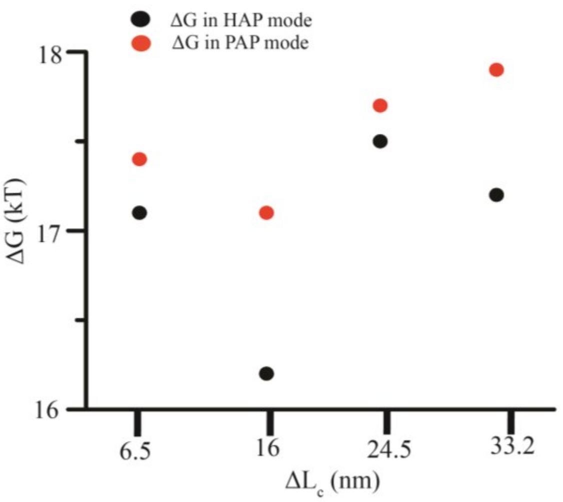

Supplementary Figure 6. Escape energy barriers for four mean extensions of Cdh23 in HAP and PAP. (In support of figure 2)

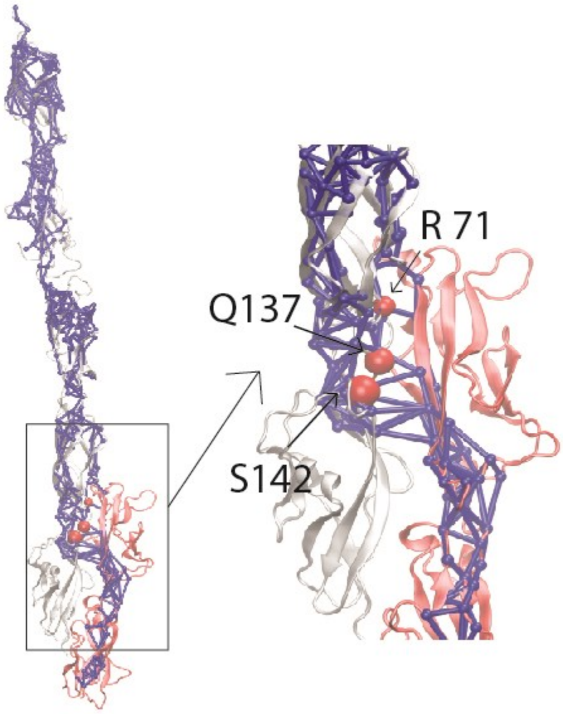

Supplementary Figure 7. Spatial positions of three major interface nodes between Cdh23 and Pcdh15. (In support of figure 3) Positions of the major interface nodes, Arginine 71, Glutamine 137, and Serine 142 between Cdh23 and Pcdh15 have been highlighted as red spheres. In the right side interfacial suboptimal paths between Cdh23 and Pcdh15 have been zoomed and all three major nodes have been marked.

Supplementary Table 1: The kinetic parameters obtained for the all unfolding steps of Cdh23 EC1-27 in PAP mode. Kinetic parameters are determined from the analysis where loading rates are calculated from the slope of the linear portion of the force curves immediate prior to unfolding or unbinding. Mean±s.d. are estimated from n=3 independent experiments.

**Extension (nm)**	**Lifetime (s) (mean±s.d.)**	**Transition state distance (x_β) (nm) (mean±s.d.)**	**Energy barrier (ΔG^*)**	$Fc\ (pN) = \Delta G^* / x_\beta$
6.4±0.4	0.026±0.004	0.073±0.002	17.1 k_BT	234.2
15.8±0.3	0.015±0.005	0.064±0.003	16.5 k_BT	257.8
24.5±0.3	0.018±0.007	0.084±0.007	16.7 k_BT	198.8
33.7±0.2	0.028±0.005	0.064±0.003	17.1 k_BT	267.1

Supplementary Table 2: The kinetic parameters obtained for the all unfolding steps of Cdh23 EC1-27 in HAP mode. Kinetic parameters are determined from the analysis where loading rates are calculated from the slope of the linear portion of the force curves immediate prior to unfolding or unbinding. Mean±s.d. are estimated from n=3 independent experiments.

**Extension (nm)**	**Lifetime (s) (mean±s.d.)**	**Transition state distance (x_β) (nm) (mean±s.d.)**	**Energy barrier (ΔG^*)**	$Fc\ (pN) = \Delta G^* / x_\beta$
6.5±0.5	0.0419±0.008	0.095±0.002	17.5 k_BT	184.2
16.9±1.4	0.0413±0.004	0.042±0.003	17.5 k_BT	416.6
24.6±0.6	0.0308±0.007	0.046±0.004	17.2 k_BT	373.9
33.2±0.9	0.0468±0.005	0.044±0.003	17.6 k_BT	401.2
