# Supplementary material for: Anisotropy in mechanical unfolding of protein upon partner-assisted pulling and handle-assisted pulling
Source: Commun Biol. 2021 Jul 29;4:925. doi: 10.1038/s42003-021-02445-y (PMC8322310; doi:10.1038/s42003-021-02445-y)
Supplement: Supplementary file 3 — Description of Additional Supplementary Files [file 42003_2021_2445_MOESM3_ESM.pdf]

Description of Additional Supplementary Files

File Name: Supplementary Data 1
Description: Supplementary data (in excel format) for the graphs in the main figures.
