# Supplementary material for: Anisotropy in mechanical unfolding of protein upon partner-assisted pulling and handle-assisted pulling
Source: Commun Biol. 2021 Jul 29;4:925. doi: 10.1038/s42003-021-02445-y (PMC8322310; doi:10.1038/s42003-021-02445-y)
Supplement: Supplementary file 5 — Reporting Summary [file 42003_2021_2445_MOESM5_ESM.pdf]

nature research

Corresponding author(s): Sabyasachi Rakshit
Jagadish Prasad Hazra

Last updated by author(s): Jun 18, 2021

Reporting Summary

Nature Research wishes to improve the reproducibility of the work that we publish. This form provides structure for consistency and transparency in reporting. For further information on Nature Research policies, see our Editorial Policies and the Editorial Policy Checklist.

Statistics

For all statistical analyses, confirm that the following items are present in the figure legend, table legend, main text, or Methods section.

n/a	Confirmed	
☐	☒	The exact sample size (n) for each experimental group/condition, given as a discrete number and unit of measurement
☐	☒	A statement on whether measurements were taken from distinct samples or whether the same sample was measured repeatedly
☒	☐	The statistical test(s) used AND whether they are one- or two-sided<br>*Only common tests should be described solely by name; describe more complex techniques in the Methods section.*
☒	☐	A description of all covariates tested
☒	☐	A description of any assumptions or corrections, such as tests of normality and adjustment for multiple comparisons
☐	☒	A full description of the statistical parameters including central tendency (e.g. means) or other basic estimates (e.g. regression coefficient) AND variation (e.g. standard deviation) or associated estimates of uncertainty (e.g. confidence intervals)
☒	☐	For null hypothesis testing, the test statistic (e.g. F, t, r) with confidence intervals, effect sizes, degrees of freedom and P value noted<br>*Give P values as exact values whenever suitable.*
☒	☐	For Bayesian analysis, information on the choice of priors and Markov chain Monte Carlo settings
☒	☐	For hierarchical and complex designs, identification of the appropriate level for tests and full reporting of outcomes
☒	☐	Estimates of effect sizes (e.g. Cohen's d, Pearson's r), indicating how they were calculated

Our web collection on statistics for biologists contains articles on many of the points above.

Software and code

Policy information about availability of computer code

Data collection	AFM data has been collected using JPK SPM desktop software provided by JPK.
Data analysis	Unfolding force curves are analyzed in home-written codes in MATLAB R2020a to determine the unfolding forces, contour lengths. Histograms are plotted using OriginPro 2018 and fitted with Kernel density estimation function.

For manuscripts utilizing custom algorithms or software that are central to the research but not yet described in published literature, software must be made available to editors and reviewers. We strongly encourage code deposition in a community repository (e.g. GitHub). See the Nature Research guidelines for submitting code & software for further information.

Data

Policy information about availability of data

All manuscripts must include a data availability statement. This statement should provide the following information, where applicable:

- Accession codes, unique identifiers, or web links for publicly available datasets
- A list of figures that have associated raw data
- A description of any restrictions on data availability

Materials and methods are written in Main manuscript file. Major data sets are available in supplementary data file. All the other data e.g. original force curves are available upon reasonable request to authors.

Field-specific reporting

Please select the one below that is the best fit for your research. If you are not sure, read the appropriate sections before making your selection.

- [x] Life sciences
- [ ] Behavioural & social sciences
- [ ] Ecological, evolutionary & environmental sciences

For a reference copy of the document with all sections, see nature.com/documents/nr-reporting-summary-flat.pdf

Life sciences study design

All studies must disclose on these points even when the disclosure is negative.

Sample size	Sample size calculation was not performed, however, the sizes were sufficiently large to reduce the errors in the estimation of most-probable parameters from force-spectroscopy studies at the single molecule level.
Data exclusions	Data exhibiting only one peak were excluded from the study as it may denote to unbinding before any unfolding. Unfolding curves exhibiting distance change more than 950 nm were excluded as the maximum stretching of Cdh23 in theoretical estimation is 950 nm.
Replication	All the reported experiments were repeated in triplicate. In every independent experiment, new set of cantilevers and protein batch were used.
Randomization	No randomization was needed.
Blinding	Blinding was not required as we have taken all the data into accounts. The analyses were done using already standardized protocols and common software.

Reporting for specific materials, systems and methods

We require information from authors about some types of materials, experimental systems and methods used in many studies. Here, indicate whether each material, system or method listed is relevant to your study. If you are not sure if a list item applies to your research, read the appropriate section before selecting a response.

Materials & experimental systems

n/a	Involved in the study	
[ ]	[x]	Antibodies
[ ]	[x]	Eukaryotic cell lines
[x]	[ ]	Palaeontology and archaeology
[x]	[ ]	Animals and other organisms
[x]	[ ]	Human research participants
[x]	[ ]	Clinical data
[x]	[ ]	Dual use research of concern

Methods

n/a	Involved in the study	
[x]	[ ]	ChIP-seq
[x]	[ ]	Flow cytometry
[x]	[ ]	MRI-based neuroimaging

Antibodies

Antibodies used	Anti-Cdh23 antobodies (PA5-43398, Thermo Fischer Scientific)
Validation	Malay et.al. 2019, Molecular Oncology, Volume13, Issue5, Pages 1092-1109. (reference from thermofisher website)

Eukaryotic cell lines

Policy information about cell lines

Cell line source(s)	Thermo Fisher Scientific - Expi-CHO cell lines
Authentication	No authentication.
Mycoplasma contamination	Tested negative by the supplier. Further, no Mycoplasma contamination is noticed.
Commonly misidentified lines (See ICLAC register)	No commonly misidentified lines.
